# A comparative analysis of algorithms for somatic SNV detection in cancer

**DOI:** 10.1093/bioinformatics/btt375

**Published:** 2013-07-09

**Authors:** Nicola D. Roberts, R. Daniel Kortschak, Wendy T. Parker, Andreas W. Schreiber, Susan Branford, Hamish S. Scott, Garique Glonek, David L. Adelson

**Affiliations:** ^1^School of Molecular and Biomedical Science and ^2^School of Mathematical Sciences, University of Adelaide, South Australia, Australia, ^3^Department of Molecular Pathology and ^4^ACRF Cancer Genomics Facility, Centre for Cancer Biology, SA Pathology, Adelaide, South Australia, Australia and ^5^School of Medicine, University of Adelaide, South Australia, Australia

## Abstract

**Motivation:** With the advent of relatively affordable high-throughput technologies, DNA sequencing of cancers is now common practice in cancer research projects and will be increasingly used in clinical practice to inform diagnosis and treatment. Somatic (cancer-only) single nucleotide variants (SNVs) are the simplest class of mutation, yet their identification in DNA sequencing data is confounded by germline polymorphisms, tumour heterogeneity and sequencing and analysis errors. Four recently published algorithms for the detection of somatic SNV sites in matched cancer–normal sequencing datasets are VarScan, SomaticSniper, JointSNVMix and Strelka. In this analysis, we apply these four SNV calling algorithms to cancer–normal Illumina exome sequencing of a chronic myeloid leukaemia (CML) patient. The candidate SNV sites returned by each algorithm are filtered to remove likely false positives, then characterized and compared to investigate the strengths and weaknesses of each SNV calling algorithm.

**Results:** Comparing the candidate SNV sets returned by VarScan, SomaticSniper, JointSNVMix2 and Strelka revealed substantial differences with respect to the number and character of sites returned; the somatic probability scores assigned to the same sites; their susceptibility to various sources of noise; and their sensitivities to low-allelic-fraction candidates.

**Availability:** Data accession number SRA081939, code at http://code.google.com/p/snv-caller-review/

**Contact:** david.adelson@adelaide.edu.au

**Supplementary information:** Supplementary data are available at *Bioinformatics* online.

## 1 INTRODUCTION

Cancer genome projects are currently working to catalogue the diversity of DNA mutations present in different cancers via high-throughput DNA sequencing of matched cancer–normal samples. These projects seek to find correlations between mutation profiles and clinical outcomes, identify mutations driving cancer progression and identify targets for novel therapeutic developments. Somatic single nucleotide variants (SNVs) are the simplest class of mutation, but their detection from matched cancer–normal sequencing data is complicated by both biological and technical noise.

Cancers arise from a single ancestral cell that has acquired enough somatic mutations to deregulate its own proliferation and expand into a large cell population with clonal mutations matching the ancestral profile. Loss of function in genes for genome stability and repair establishes a mutator phenotype in which a greater rate of somatic mutation provides the cell population with a vast repository of mutations in low copy. Those mutations conferring a selective advantage preferentially expand in number, forming subpopulations with the same subclonal mutations. Clonal and subclonal mutations include drivers of the cancer phenotype, and passenger mutations that arose in the same clonally expanding cell line as a driver (Lee and Swanton, 2012; Loeb, 2011; Salk *et al.*, 2010).

Analysis of cancer sequencing data has unique challenges, including: methods for analysing matched cancer–normal samples to distinguish germline polymorphism from somatic variation; genome rearrangements that do not align well to the reference; and cancer sample heterogeneity from subclonal variation and sample impurity (Ding *et al.*, 2010; Gundry and Vijg, 2012; Meyerson *et al.*, 2010).

In addition to this biological complexity are several sources of mapping and sequencing error, both random and systematic. Error profile assessments of the Illumina sequencing platform have identified increased error rates at read ends, a tendency towards transversion base-call errors, a low indel error rate and systematic sequence-specific errors following inverted repeat sequences and GG motifs (Dohm *et al.*, 2008; Meacham *et al.*, 2011; Nakamura *et al.*, 2011). The majority of systematic errors assessed by Meacham *et al.* (2011) only had base-call errors on one of the two DNA strands. They suggested the presence of different motifs immediately preceding a certain site during sequencing on different strands leaves one strand significantly more prone to phasing error than the other. Greater depth of sequencing is often advanced as the solution to separating sequencing errors from real variation, but the susceptibility of particular sites to recurrent base-call errors is consistently observed at any depth. Furthermore, increased depth has the unintended side effect of allowing sites with lower susceptibility to systematic error to accumulate multiple such mistakes. This is a particularly significant problem in cancer sequencing, as subclonal variation and sample impurity give rise to mutations at the same low allelic fractions as aggregations of systematic error.

## 2 SOMATIC SNV DETECTION

Somatic SNV detection is a preliminary step in most cancer sequencing projects, feeding into various downstream analyses addressing the broader goals of cancer genome research. How successfully the motivating research goals are met depends on the quality of the mutation set used as input for further work.

Early publications, such as the malignant melanoma cell line analysis by Pleasance *et al.* (2010), relied on independent genotype calling of the two samples followed by subtraction of the normal sample calls from the cancer calls to obtain a candidate somatic mutation set. However, this ‘subtraction’ method using standard algorithms for SNV calling in single samples did not optimize the detection of shared germline polymorphisms by jointly analysing the two samples, nor were standard genotyping algorithms designed to detect variants at the low allelic fractions found in cancer samples.

Four SNV calling algorithms specifically designed for joint analysis of matched cancer–normal samples are VarScan (Koboldt *et al.*, 2009, 2012), SomaticSniper (Larson *et al.*, 2012), JointSNVMix (Roth *et al.*, 2012) and Strelka (Saunders *et al.*, 2012). We also note the availability of MuTect (Cibulskis *et al.*, 2013); however, this tool was unpublished at the time of the comparison presented here.

### 2.1 Variant calling algorithms

A comparison of the underlying models and methods used by these four SNV calling algorithms is provided in Supplementary Table S1. It should be noted that these SNV calling algorithms do not model aneuploidy or copy number variation, and SNV calls will be confounded with any such events.

#### 2.1.1 VarScan2

VarScan2 first independently analyses pileup files from the cancer and normal samples to heuristically call a genotype at positions achieving certain thresholds of coverage and quality. If there is a variant base constituting at least the ‘minimum variant frequency’ of all reads (0.20 by default), then the genotype is called as either heterozygous (variant <75%) or homozygous variant (variant >75%). Otherwise, the site is deemed homozygous reference. Then, at positions where these heuristically determined genotypes do not match in cancer and normal, a one-tailed Fisher’s exact test is performed on the read counts. A significant result is called somatic if the normal sample was homozygous reference, or as loss-of-heterozygosity (LOH) if the normal sample was heterozygous, or unknown if the normal sample was called homozygous variant and the cancer sample did not match. VarScan2 can take optional purity estimates for the cancer and/or normal sample, and will adjust its variant frequency thresholds accordingly.

#### 2.1.2 SomaticSniper

SomaticSniper uses a Bayesian probability calculation to estimate the posterior probability of each possible joint genotype across the normal and cancer samples given the data observed and prior genotype likelihoods based on the reference sequence, sequencing error rate, population mutation rate and somatic mutation rate. Each site is given a ‘somatic score’ (S), a phred-scaled posterior probability that the cancer and normal genotypes are the same.

#### 2.1.3 JointSNVMix2

JointSNVMix is based on a different Bayesian approach using a mixed binomial model. The model considers each site across the two samples to have one of nine joint genotypes possible after reducing the bases to ‘A’ for a reference and ‘B’ for a variant base. The set of joint genotypes across all sites is considered to have a multinomial distribution whose parameters have a Dirichlet prior with trained hyper-parameters. At each site, the number of reads supporting the reference base in cancer and normal is considered to have a binomial distribution. The binomial probability parameter is modelled from a beta prior with trained hyper-parameters, conditional on the joint genotype.

First, the hyper-parameters of the multinomial and binomial distributions are trained by expectation maximization over a subset of the data, using set default values as priors. Then, the posterior probability of each joint genotype is calculated at each site. The basic model, JointSNVMix1, assumes the data have perfect base calls and alignment, whereas the extended model, JointSNVMix2 (JSM2), weighs each read by base and mapping quality.

#### 2.1.4 Strelka

Strelka performs an initial realignment around indels in the normal and cancer BAM files, then uses a complex set of calculations, again based on a Bayesian probability model, to report the most likely genotype at candidate sites along with the phred-scaled joint probability of the most likely normal sample genotype and the event of any somatic mutation in the cancer sample. The normal sample allele frequencies are modelled as diploid genotypes with a noise term to factor in sequencing and mapping errors, whereas the cancer sample allele frequencies are modelled as a mixture of the normal sample and somatic variation at any frequency. The exact details of the model, which are too numerous to describe here, take into account base and mapping qualities, strand bias, the prior probability of any site being a somatic mutation and the expected rate of heterozygosity in the normal sample.

### 2.2 Filtering candidate SNV sites

These somatic SNV calling algorithms use a variety of information to calculate a somatic probability score for each candidate site returned. However, the full complexity of noise present in any DNA sequencing dataset is not typically considered during SNV calling, so raw output sets of candidate SNV sites typically require filtering to remove likely false positives.

Koboldt *et al.* (2012) recommended various filters addressing the quality of variant bases at candidate sites, including within-read position, extreme strand bias, flanking homopolymer motifs and low mapping quality. Larson *et al.* (2012) recommended filtering on the basis of strand bias, mapping quality, proximity to read ends, proximity to indels, nearby homopolymers, nearby SNVs and depth. Unlike the other algorithms that solely provide SNV calling capability, Strelka has its own inbuilt post-calling filter for sites with extremely high depth indicative of over-mapping to repetitive sequences; reads with too many mismatches to the reference; and the presence of spanning deletions mapped across the site. Strelka outputs the candidates identified both before and after the post-calling filter, so either set is available for analysis (Saunders *et al.*, 2012).

## 3 RESULTS

VarScan2, SomaticSniper, JSM2 and Strelka were used to return candidate SNV sites from matched cancer–normal exomes from a chronic myeloid leukaemia (CML) patient, sequenced with the Illumina HiSeq platform in paired 100 bp reads (SRA081939). The algorithms were also applied to similar exome sequencing data from non-cancerous patient samples (randomly split into two equal-sized subsamples to mimic production of two normal sequencing samples from each individual) to assess the output returned in the absence of any real somatic variation between samples. Details of the data and methods used are available in the Supplementary Information.

### 3.1 Raw output

The output from each of the four algorithms includes both gain-of-variation somatic SNV candidates and sites of apparent LOH in the cancer sample. Using low probability score thresholds for inclusion to generate large candidate sets, the raw output consisted of 2667 somatic and 1720 LOH VarScan candidates; 2663 somatic and 175 LOH SomaticSniper candidates; 2178 somatic and 2040 LOH JSM2 candidates; and 438 somatic and 29 LOH Strelka candidates. Only 50 of the somatic candidates were returned by all four callers, and the vast majority of sites were uniquely returned by one caller.

In keeping with the primary purpose of these algorithms, we chose to focus this review on the somatic candidates rather than LOH. A short discussion on the LOH candidates returned by these algorithms is included in Section 4.1.

As expected, given the low probability stringency used to define candidate inclusion, the number of sites returned exceeded the number of expected true positives by orders of magnitude. Two simple approaches for reducing the number of candidates would be to remove sites returned with a low probability score, or to remove candidates uniquely identified by one caller.

**Fig. 1.**
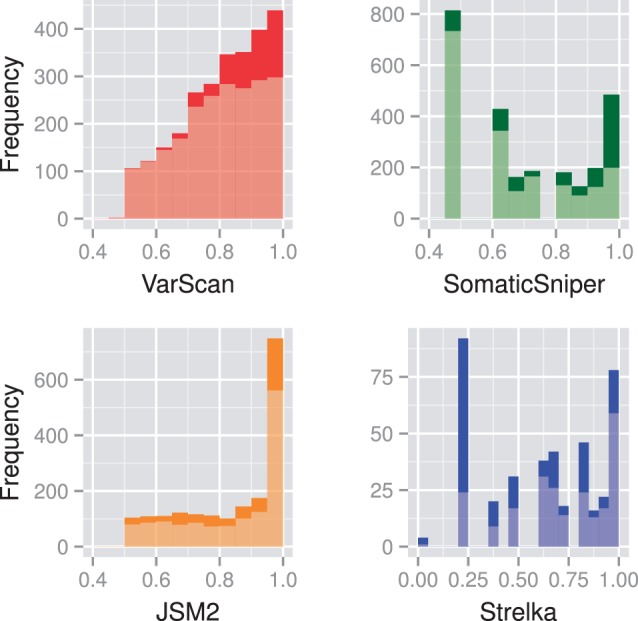
Frequency distribution of probability scores for somatic candidates in the raw output from the CML exome, with sites unique to each caller in a light shade and sites returned by multiple callers in a dark shade. Note that gaps between SomaticSniper and Strelka frequency peaks are an artefact due to the phred scaling used by these tools

**Fig. 2.**
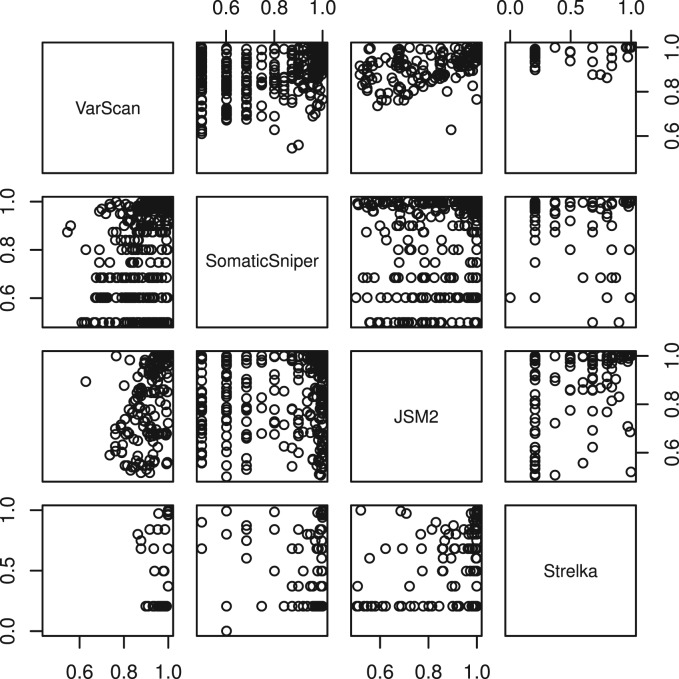
Probability scores of somatic candidates in common between pairs of algorithms for the CML exome. Pearson correlation coefficients between pairs are VS&SS 0.50, VS&JS 0.59, VS&ST 0.42, SS&JS 0.23, SS&ST 0.21 and JS&ST 0.46

The distribution of somatic probability scores for sites unique to each caller and returned by multiple callers, as shown in Figure 1, indicates that filtering out sites returned by only one algorithm would remove sites regarded to have high somatic probability by one such measure. Furthermore, Figure 2 shows that many sites returned with a high probability score by one caller are returned with a much lower probability score by another. If the first step of post-calling filtration was to remove from each caller’s output the sites returned with low probability, then information on sites being found by multiple callers but at markedly different probabilities would be lost. Given the poor correlations between probability scores from different callers for the same sites, their intrinsic value is questionable.

Observable in both Figures 1 and 2 is that, unlike the other callers, none of VarScan’s lowest probability candidates were returned by any other caller. In sharp contrast, many of Strelka’s candidates at probability 0.20 were returned with high probability scores by other callers. The somatic probabilities in the raw JSM2 output are calibrated far too high, with 337 somatic candidates returned with probability score 1.0, and a further 748 somatic candidates returned with probability scores between 0.95 and 1.0, when the expected number of true positives is closer to 100.

### 3.2 Filters

Rather than reducing the number of candidates by increasing the probability score threshold for inclusion, we applied filters designed to remove sequencing and mapping errors. Sites were removed if any of the following criteria were met (considering variant bases in the cancer sample for somatic candidates and variant bases in the normal sample for LOH candidates): variant bases emanate exclusively from one strand; mean variant base quality is less than 15; no variant has base quality over 30; mean variant mapping quality is less than 15; no variant has mapping quality over 40; more than two candidate SNVs (identified by any algorithm) are within 50 bp either side; spanning deletions contribute >20% to the overall depth in either sample; or candidates are immediately adjacent to indels in >20% of reads in either sample. A detailed breakdown of filtering results is available in the Supplementary Information.

By far the most significant filtering metric was the requirement to have at least one relevant variant base on each strand, with 5437 out of 10 438 candidates removed for having 100% strand bias. Although this filter was designed to remove systematic sequencing errors, complete strand bias can occur solely as a result of random sampling between the two strands, especially at low depths. For a site with a total of *v* variant reads, the chance that all of those reads occur on one strand is 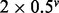. Using these per-site strand bias probabilities for this dataset, the expected number of sites with 100% strand bias is 1272, less than a quarter of the number actually observed. When applying the same methods to whole genome sequencing cancer–normal data (not shown), the expected number of sites with 100% strand bias was less than half the number observed. The relative extent of strand bias may be greater in exome sequencing because of the exome capture design tending to cover sequences just outside the targeted regions from one direction (and one strand) only. However, the profusion of strand biased variants in both exome and whole genome data supports the descriptions of systematic errors of sequencing by Nakamura *et al.* (2011) and Meacham *et al.* (2011).

There were significant differences between the filter pass rates of the four output sets, as presented in Table 1. The raw output from Strelka was least susceptible to these indicators of sequencing and mapping error, while the output from JSM2 was most susceptible.

**Table 1.** Pass rates (%) of candidate sites (somatic and LOH) through the strand bias filter and the combination of all other filters

Algorithm name	Strand bias	All other filters
VarScan	50.3	58.3
SomaticSniper	50.4	66.2
JSM2	45.8	47.9
Strelka	70.9	89.9

The combined filters for variant base and mapping quality, nearby SNVs, spanning deletions and adjacent indels were applied *after* the removal of sites with 100% strand bias. All differences are significant, except between VarScan and SomaticSniper with the strand bias filter.

### 3.3 Comparison and characterization of candidate sites

**Fig. 3.**
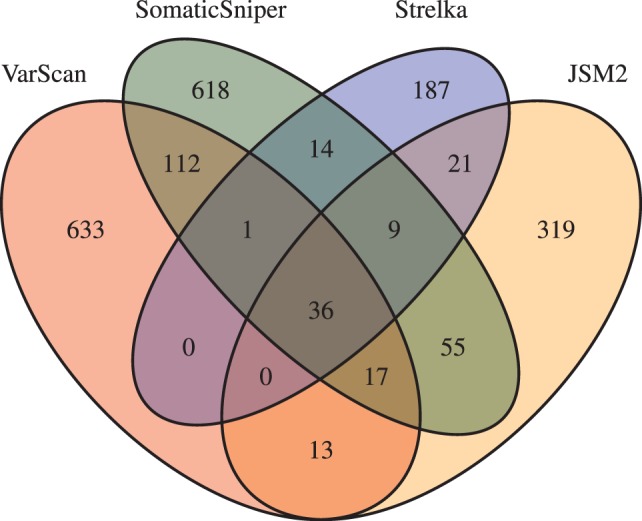
Overlaps between somatic SNV candidate sets in the filtered output for the CML exome

After filtering, 2920 candidate sites remained, including 812 somatic and 475 LOH VarScan candidates; 862 somatic and 85 LOH SomaticSniper candidates; 470 somatic and 455 LOH JSM2 candidates; and 268 somatic and 28 LOH Strelka candidates, with overlaps between the four sets of somatic candidates illustrated in Figure 3.

**Fig. 4.**
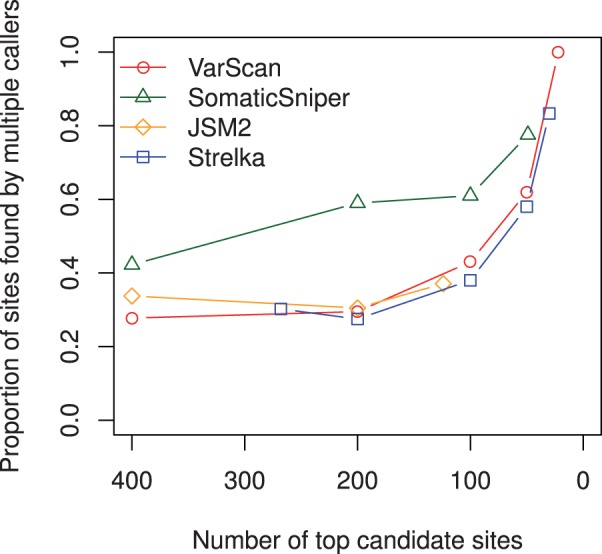
Proportion of somatic sites found by multiple callers as the probability score threshold of each caller is increased to 1.0 and the number of candidate sites reduces

**Fig. 5.**
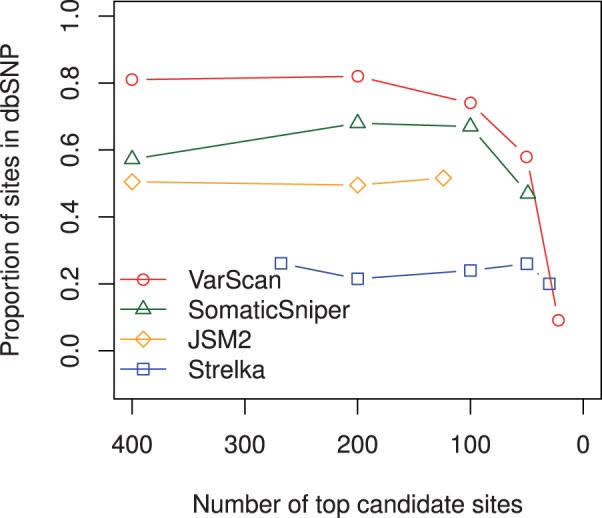
Proportion of somatic candidates present in dbSNP as the probability score threshold of each caller is increased to 1.0 and the number of candidate sites reduces

Figures 4 and 5 present some effects of increasing each algorithm’s somatic probability score threshold for inclusion to one, and thus reducing the number of candidate sites in their output to the top calls.

Figure 4 illustrates that the proportion of somatic sites found by any other caller (at any probability threshold) improves as the candidate sets are reduced to their top calls. VarScan’s top 22 somatic candidates were all returned with probability 1.00 by all four callers. Of SomaticSniper’s top 49 candidates, 11 were not returned at any probability level by the other three algorithms, while of JSM2’s top 124 candidates, 78 were not returned by any other caller. Strelka’s top 30 candidate sites included 25 returned by the other three callers with probability 1.00, except for two sites below a 20% variant frequency missed completely by VarScan. The five sites given 1.00 somatic probability by Strelka but not returned at any probability level by the other three algorithms were low-allelic-fraction candidates with variant proportion in the normal sample from 0.0 to 1.5% and in the cancer sample from 2.4 to 5.5%.

Figure 5 illustrates the proportion of somatic sites present in dbSNP as the output sets are reduced to their top calls. Candidate somatic mutation sites found in dbSNP are sometimes interpreted as germline polymorphism false positives (Roth *et al.*, 2012), or sites of common sequencing error contaminating the database. This is not always true, as some sites within dbSNP have validated as true-positive somatic mutations in cancer. While no particular site should be removed as a false positive solely on the basis of a dbSNP entry, the overall proportion of sites in dbSNP should still be a valid indicator of the comparative number of germline false positives in different output sets. When considering more than 100 candidates, VarScan appears to be the worst offender with ∼80% constituency of dbSNP compared with SomaticSniper ∼60% and JSM2 ∼50%. This vastly improves within the top set of 22 VarScan somatic candidates and mildly improves within the top set of 49 SomaticSniper candidates. As JSM2 considerably overestimates its somatic probability scores, its top tier of somatic calls can not be reduced to any <124, and no improvement is seen. In contrast, only ∼20% of Strelka’s candidate sites at any probability cut-off are present in dbSNP, indicating Strelka is not as prone to returning germline polymorphisms. Rather, Strelka’s false positives may tend to be the result of sequencing errors.

**Fig. 6.**
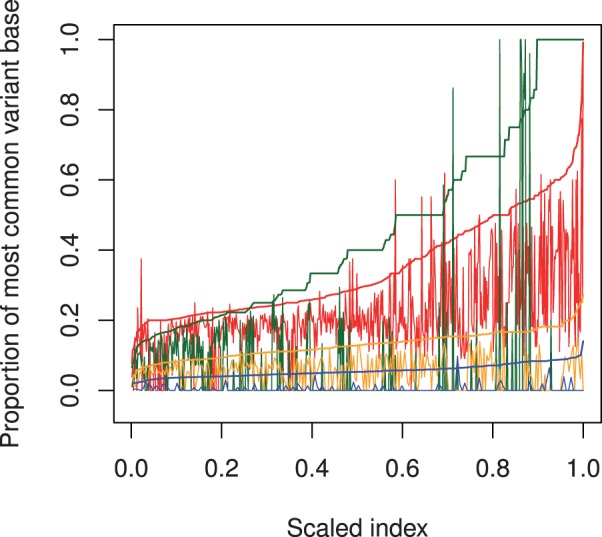
The proportion of total depth contributed by the most common variant base in the cancer (smooth lines) and normal (jagged lines) for somatic sites uniquely returned by VarScan (red), SomaticSniper (green), JSM2 (orange) and Strelka (blue). The horizontal axis is the scaled index of each site after sorting by variant proportion in the cancer (scaled index chosen for comparisons across different sample sizes)

The characteristics of somatic candidates uniquely returned by each caller are illustrated by Figure 6. Each set of sites returned solely by one caller was sorted by variant proportion in the cancer sample. Then the variant proportion in the cancer (smooth lines) and normal sample (jagged lines) were plotted against the scaled index of each site.

VarScan’s default algorithm design returns somatic candidates if, for variant bases above 15 in quality, the cancer variant proportion is between 20 and 75% and the normal variant proportion is outside this range. Although obscured to some extent in Figure 6, which plots the proportion of all variant bases not just those above 15 in quality, it is apparent that sites uniquely returned by VarScan are those at which the variant proportion in the normal sample falls slightly below 20% (and above in the cancer sample). The vast majority of unique VarScan candidates had a strong variant signal in both samples, again suggesting a tendency for VarScan to return germline polymorphism false positives.

SomaticSniper shows a distinctive discretized quality in its cancer variant proportions, explained by the low depth at most sites uniquely found by SomaticSniper. Unlike the other callers, SomaticSniper had no lower bound for the depth of its candidate sites, and 70% of the candidates unique to SomaticSniper had depths in the cancer sample below 12. At low depths, variant allele frequencies can only occur at certain discrete levels, largely explaining the pattern in Figure 6. While the sites at the discretized variant frequencies and low depths appear to have no corresponding variant signal in the normal sample, yielding the result that 71% of sites unique to SomaticSniper have no variant signal in the normal, SomaticSniper’s output also contained unique sites at higher depths with variant signals in evidence from both samples, constituting likely germline polymorphism false positives.

JSM2 uniquely returned low-allelic-fraction candidates with variant proportions in the cancer ∼5–20%, but variant signals in the normal sample were also present at 75% of these sites. Half of the candidates unique to JSM2 had depths in the cancer sample above 90. This suggests a possible weakness of JSM2: that at high depths of sequencing, a *statistically* significant difference between variant proportions in the two samples is more likely, regardless of the biological significance.

Strelka appears tuned to detect even lower allelic fractions, as its unique calls have variant proportion in the cancer around 5%. In contrast to JSM2, 72% of Strelka’s unique output had no variant base in the normal sample.

### 3.4 Non-cancer exomes

For each of two exomes sequenced to high depth from non-cancerous samples, we twice randomly split the BAM file in half and applied the somatic calling algorithms to these matched normal–normal samples to return purely false-positive candidate sets. After applying the previously described post-calling filters, the number of false-positive sites output by all four algorithms in the four cases were 5, 7, 10 and 11. On inspection, these 33 false positives were seemingly indistinguishable from some of the somatic candidates identified in the cancer samples, with depths ranging from 10 to 73 and most having somatic probability scores above 0.90.

## 4 CONCLUSIONS AND FUTURE PERSPECTIVES

Comparing the candidate SNV sets returned by VarScan, SomaticSniper, JSM2 and Strelka revealed substantial differences as to the number and character of sites returned; the somatic probability scores assigned to the same sites; their susceptibility to various sources of noise; and in their differing sensitivities to candidate mutations at a low allelic fraction.

### 4.1 LOH candidates

All four algorithms return candidate LOH events implying loss of variation in the cancer sample. The term typically implies a deletion event, but duplication events also register as LOH candidates when a previously 1:1 heterozygous signal shifts to a 2:1 signal in the duplicated region. LOH candidates may also arise from somatic mutations present in the ‘normal’ cell sample that were never present as germline polymorphisms in the general cell population of the individual.

Given these confounding factors, the LOH candidates returned by these tools are not, by themselves, a strong indication of actual LOH. These SNV calling algorithms have not been optimized to detect LOH regions, and merely give the output as a by-product of their main purpose. Regions with a relatively high frequency of LOH candidates may indicate real copy number variation, but these should be investigated by alternative means like aCGH.

After filtering, the proportion of LOH candidates in the output from each algorithm was ∼50% for JSM2, 40% for VarScan and 10% for SomaticSniper and Strelka. As regions of interest with relatively high frequency of LOH candidates are still identifiable in smaller output sets, and as the LOH candidates assigned the highest probability scores by VarScan also tend to be returned by SomaticSniper and, to a slightly lesser extent, by Strelka as well, no obvious benefit is gained in using VarScan or JointSNVMix to return greater numbers of LOH candidates.

### 4.2 Somatic candidates

The main purpose of the four algorithms analysed here is the detection of candidate somatic SNV sites purporting gain of variation in the cancer sample. A core group of sites was identified by all algorithms at high probabilities, though given the identification of a few similar sites within the non-cancerous exomes, false positives are likely to be included even in this common set. Beyond this core group, there were marked differences in the probability scores assigned to the same sites and in the characteristics of sites returned by one algorithm only.

VarScan (Koboldt *et al.*, 2009, 2012) has the advantage that its top set of somatic candidate sites are convincing mutations with high concordance with other callers. However, aside from these relatively clearcut candidates identified by all calling algorithms, VarScan’s output at lower probability thresholds appear inundated by germline polymorphism false positives, an understandable consequence of VarScan’s algorithm design. By automatically classifying sites with 20–75% variant frequency as heterozygous and sites just outside these thresholds as homozygous, any germline polymorphism that, by chance and the vagaries of sequencing bias, registers either side of these cut-offs in the two samples becomes a candidate for Fisher’s test. At reasonably high depth, even a small difference in variant proportions can achieve statistical significance, regardless of biological significance. Aside from the high rate of germline false positives, the other major drawback to VarScan is its inability to detect low-allelic-fraction candidates below its minimum variant frequency for heuristic genotyping. We used the default settings with the lower bound of 20% and thus VarScan failed to return even the most convincing mutations below this level. Given the paucity of convincing candidates returned by VarScan that would not also be returned by SomaticSniper or Strelka at high probability, we conclude little benefit is gained in running VarScan as opposed to these other algorithms.

SomaticSniper (Larson *et al.*, 2012) has no minimum depth requirements and thus is liable to return a unique set of candidates at the low depths that surround target regions in an exome capture assay. If desired, it would be a simple matter to add an additional post-calling filter for minimum depth. Apart from those sites identified at low depths in the exome sample, other sites uniquely found by SomaticSniper tended to be mutations with a 10–30% allelic fraction. As a means of generating a variety of candidate SNV sites without any particular drawbacks, SomaticSniper is a practical and credible program, though its results should by no means be interpreted as constituting an inherently true mutation profile.

JSM2 (Roth *et al.*, 2012), in its current implementation, is extremely inconvenient to use. Unlike the other algorithms, which only output sites of interest above particular, customizable thresholds, JSM2 includes a line in the output file for every single site in the input BAM files regardless of their somatic or LOH probability scores. This high volume of output is unnecessarily awkward for exome data and impracticable for whole genome data. Not only are the training and classifying steps of JSM2 considerably slower than either SomaticSniper or Strelka, but it is also left up to the user to develop a reasonable method for extracting the candidate sites. Even after the raw output was pared back to genuine sites of interest, JSM2’s candidates had the lowest pass rate through filters to remove sequencing error and then after that were found to have 50% constituency in dbSNP. These results suggest JSM2 candidates are vulnerable to false positives from both sequencing error and germline polymorphisms. Furthermore, JSM2 vastly overestimates the somatic probabilities assigned to each site, such that a small set of the highest ranking candidates can not be derived. This problem would be overcome by lowering the prior parameter for expected rate of somatic mutation, but in this analysis, only the default settings were considered. The sites uniquely returned by JSM2 have a 5–20% allelic fraction in the cancer, with the majority having only slightly lower variant signals in the normal sample. We suggest that the extra sites identified by JSM2 do not, on current evidence, include enough convincing candidates to outweigh the inconvenience of its unwieldy implementation.

Strelka (Saunders *et al.*, 2012) is a computationally efficient program that returns a much smaller output set of candidates than the other algorithms. Some Strelka calls with probability scores of 0.0 and 0.2 overlapped with high probability output from other callers, so all Strelka sites were considered valid candidates for analysis, regardless of probability score. The sites uniquely found by Strelka were low-allelic-fraction mutations ∼5% variant frequency in the cancer. Given the clinical importance of subclonal mutations with drug resistant capability, Strelka is a valuable tool for identifying candidate mutations below the detection level of other algorithms, without returning an excessive number of dubious results. Strelka also identifies the same high-probability clonal-type mutations as the other algorithms. Of the four algorithms investigated here, we consider Strelka and SomaticSniper to be the best overall performers.

Although all designed for the same, conceptually simple, task of identifying candidate somatic SNV sites in matched cancer–normal sequencing data, the complexity of the systematic noise permeating sequencing data affects each algorithm in different ways. In practice, many cancer sequencing projects have relied on one SNV calling pipeline to generate candidates. The poor consensus between different algorithms beyond a small group of clearcut candidates suggests extreme care is needed to prevent over-interpreting any one output set as being intrinsically representative of the true mutation profile. Using two algorithms with different vulnerabilities to error that appear ‘tuned’ to detect different types of candidates is likely to provide a significant reduction in false negatives, at the cost of returning more false positives. However, as post-calling filters can be designed to remove as many unconvincing candidates as desired, the benefits of seeking alternative candidate sites from different algorithms still hold.

Given the significant differences and contradictions in the output returned by each SNV calling algorithm, extreme caution should be applied when interpreting their somatic probability scores, choosing sites for subsequent analysis, and reporting results. Over-interpreting the output from any one SNV calling algorithm as being the closest possible estimation to the real set of somatic SNVs risks the inclusion of errors that algorithm is vulnerable to, and the omission of real mutation identifiable by different algorithm designs.

### 4.3 Filters, ranking and assessment of candidate sites

Candidate SNV sets are typically filtered to remove multiple indicators of sequencing error. Incorporating a knowledge of the sequencing platform’s error profile *after* initial SNV calling, rather than including it as part of the calling algorithm, allows the basic calling algorithms to remain relevant for various iterations of the sequencing technology. As the error profiles of DNA sequencing platforms rapidly shift with every technical upgrade, it makes more sense to continually update a set of best-practice filters than redesign the calling algorithms every year. Having said this, there is currently no best-practice filtering method commonly agreed with.

Aside from the six features we used for filtering (strand bias, nearby SNVs, spanning deletions, adjacent indels, variant base and mapping qualities), some other filters recommended in the literature include within-read position and the presence of particular surrounding sequence motifs (Koboldt *et al.*, 2012; Larson *et al.*, 2012; Meacham *et al.*, 2011; Nakamura *et al.*, 2011). Errors are known to accumulate in and around homopolymers, inverted repeats and G-rich motifs such as GGT and GGC, so these would be ideal inclusions in a best-practice filtering design for Illumina data. The simplest and most accessible method of filtering is the independent definition of set cut-offs for different features dictating the inclusion or exclusion of each site. However, as more error features are described and included in filtering, this simple method becomes less appropriate. For example, while it may be reasonable to require a certain minimum mapping quality for every candidate, it would not be reasonable to remove every candidate site following a GG motif, even though this too has been strongly associated with error. A more sophisticated method of filtering out likely false positives would be the joint consideration of multiple error indicators (including surrounding motifs) in the definition of a classification or probability recalibration rule reflecting the gamut of features known to correlate with error. Training such a rule requires an extensive set of validated true- and false-positive SNV candidates within data from the sequencing platform of interest, so is most practical at large sequencing centres.

Metrics used to gauge the quality of candidate sites after filtering include the proportion of sites in dbSNP or HapMap to assess germline polymorphism false positives, and pass rate through filters to assess sequencing error false positives. In general, such metrics are difficult to interpret because a low rate of one false-positive type may imply either a high true-positive rate or a high false-positive rate of the other type. Another metric often used to assess candidate SNV sets is their transition–transversion ratio, under the assumption that departure from the observed Ti–Tv ratios of SNPs in normal genomes indicates contamination from sequencing error false positives. However, the many reports of cancers presenting with transition–transversion ratios significantly different from the typical levels within non-cancerous genomes (Liu *et al.*, 2002; Oki *et al.*, 2009; Yang *et al.*, 2003), coupled with the fact that germline polymorphism false positives naturally correspond to the standard ratios, suggest the transition–transversion ratio is a poor metric for assessing cancerous somatic mutations.

Although deletion and duplication events confound the search for SNV candidates, at present these SNV calling algorithms only model diploid variation. Information on copy number variation from aCGH or other methods should be kept in mind when interpreting output from SNV calling algorithms.

### 4.4 Understanding the molecular basis of cancer

Cancer genome projects seek to identify mutations of therapeutic or biological interest, and to correlate mutation profiles with treatment outcomes for the development of personalized medicine and improved cancer survival rates. Somatic SNVs are an integral part of the mutational landscape, and, with an understanding of their relative strengths and weaknesses, a logical selection of multiple SNV calling algorithms should be used to offset their individual quirks and reduce the number of false negatives. As extensive validation experiments are often impractical, post-calling filters designed to remove sequencing and analysis errors can reduce the burden of excessive candidates as much as required before further investigation.

Although some number of false positives is inevitable in any candidate mutation set, if the patient cohort is large enough, relevant and actionable mutations will be ultimately identified by their recurrence in multiple patients. This is slightly confounded by the presence of systematic sequencing errors accumulating at susceptible genomic positions across different sequencing samples, but these should balance out between patients with different clinical outcomes and have little effect on correlation analysis. However, for rare cancer subtypes for which large patient cohorts are difficult to recruit, additional validation steps may be needed to prevent false positives contaminating the data and obfuscating the associations between mutational profiles and clinical outcomes.

*Funding*: National Health and Medical Research Council of Australia (1027531 to S.B., H.S.S. and D.L.A., and fellowship 1023059 to H.S.S.).

*Conflict of Interest*: none declared.

## Supplementary Material

Supplementary Data
