# Supplementary material for: A comparative analysis of algorithms for somatic SNV detection in cancer
Source: Bioinformatics. 2013 Jul 9;29(18):2223–30. doi: 10.1093/bioinformatics/btt375 (PMC3753564; doi:10.1093/bioinformatics/btt375)
Supplement: Supplementary Data [file supp_29_18_2223__index.html]

A Comparative Analysis of Algorithms for Somatic SNV Detection in Cancer — A comparative analysis of algorithms for somatic SNV detection in cancer — A comparative analysis of algorithms for somatic SNV detection in cancer — Supplementary Data 

# A comparative analysis of algorithms for somatic SNV detection in cancer

## 

files

**Files in this Data Supplement:**

- Supplementary Data - pdf file
