# Supplementary material for: A comparative analysis of algorithms for somatic SNV detection in cancer
Source: Bioinformatics. 2013 Jul 9;29(18):2223–30. doi: 10.1093/bioinformatics/btt375 (PMC3753564; doi:10.1093/bioinformatics/btt375)
Supplement: Supplementary Data [file supp_btt375_SuppInfo.pdf]

Table 1: Overview of somatic SNV calling algorithms

	**VarScan2**	**Somatic-Sniper**	**JointSNVMix2**	**Strelka**
Input	tumour and normal mpileups	tumour and normal BAMs, reference fasta	tumour and normal BAMs, reference fasta, parameter file (from `train`)	tumour and normal BAMs, reference fasta, aligner-specific config file
Output format	custom or VCF	custom or VCF	custom	VCF
Genotype model	heuristic variant frequency thresholds	Bayesian model of diploid genotypes based on MAQ	Bayesian model of diploid genotypes using binomial mixture model	Bayesian model of noisy diploid normal sample and tumour mixture
Probability score	Fisher's exact test p-value	Phred scaled somatic score	posterior probability of every joint genotype	Phred scaled somatic score for the most likely normal genotype
Base quality	considers bases above 15 in quality	via MAQ algorithm	bases weighted by quality	included in SNV likelihood
Mapping quality	NA. Users can set a minimum when writing pileup files	via MAQ algorithm. Option to set a minimum.	reads weighted by quality	considers two tiers of mapping quality
Coverage	minimum 8 reads	no minimum coverage	training step uses minimum 10 reads	no minimum coverage
Sequencing error rate	NA	via MAQ algorithm	NA	included in noise term
Somatic mutation rate	NA	optional, with default 10^{-2} (we used 10^{-4})	dependent on prior parameters	set to 10^{-6}
Heterozygosity rate	NA	default is 10^{-3} (human)	dependent on prior parameters	set to 10^{-3}

1 Data and methods

1.1 DNA sequencing data

The study was performed in accordance with the Declaration of Helsinki and approvals were obtained from the relevant institutional review boards.

Whole exome sequencing (Illumina TruSeq Exome Enrichment) was performed on matched cancer (HSS41; bone marrow mononuclear cells, 100% leukemic, obtained at diagnosis) and non-cancer samples (HSS43; cultured bone marrow mesenchymal stem cells) of a 54 year old female chronic myeloid leukemia patient. We obtained 100 bp paired-end reads with the Illumina HiSeq at a median depth of 78× in the cancer and 75× in the normal (within the targeted exome capture regions). The sequencing data was aligned to build 37 of the human genome using BWA and realigned around small indels using the GATK IndelRealigner. The accession number for this data is SRA081939.

Similar Illumina exome sequencing datasets were also obtained from two non-cancerous patients. Sample HSS34 was from a patient with familial ACTH-independent macronodular

adrenal hyperplasia and sample HSS52 was from a patient with familial hyperaldosteronism. Go code to randomly assign each read pair in a name-sorted BAM file to one of two new output BAM files (see Appendix A.1) was run twice for both HSS34 and HSS52, and then Samtools 0.1.18 (Li et al. 2009) was used to re-sort and re-index.

For this analysis, we used the build 37 reference sequence `hs37d5.fa`[1].

1.2 Somatic SNV calling

We searched for SNV sites using VarScan 2.2.11 (Koboldt et al. 2009, 2012), Strelka 0.4.6.2 (Saunders et al. 2012), JointSNVMix2 0.7.5 (Roth et al. 2012), and SomaticSniper 1.0.0 (Larson et al. 2012). See Appendix A.2.

VarScan requires input in pileup format, generated with Samtools' `mpileup` command using a minimum mapping quality of one. We ran VarScan's Fisher test with a 0.50 significance level to return a large number of candidate sites for comparison with other programs' outputs.

We ran SomaticSniper with minimum mapping quality of one, a prior probability for somatic mutation of 0.001 and minimum phred-scaled somatic score threshold of three (equivalent to a 0.499 probability score).

The training step of JointSNVMix2 considers every 100th site with a minimum depth of ten in both cancer and normal samples. We used the default prior parameters for training and the trained parameters for the final classification. JSM returns output information for every site in the input BAM files with no option to limit this to the most likely candidate sites. To reduce the size of the output, we first discarded sites with more than 50% reported probability of being homozygous reference in both cancer and normal. Candidate sites were then defined as those with minimum depth of ten and more than 50% reported probability of being a somatic or LOH mutation.

We ran Strelka using the BWA version of the configuration file, turning off the filter to remove variants at read depth greater than three times the chromosomal mean as is recommended for exome sequencing where extremely variable depth is expected. To simplify subsequent analyses, we removed the small number of output lines with no base listed in the variant field but did not filter out sites returned with somatic likelihood less than 50% as the number of candidate sites returned by Strelka was already much less than those output by other algorithms.

1.3 Collecting and reformatting data on candidate sites

To compare the candidate somatic SNV sites returned, we developed a procedure to extract meaningful information on each candidate site and save it in a relatively simple format for comparative analysis.

For each sample, we saved a list of the candidate site positions identified by the four SNV calling algorithms and generated an extended pileup file containing the depth, sequence of

[1] `ftp://ftp.1000genomes.ebi.ac.uk/vol1/ftp/technical/reference/phase2_reference_assembly_sequence/hs37d5.fa.gz`

raw base calls, base qualities, mapping qualities and within-read positions of bases mapped to each candidate site[2]. We ran `cleanpileup.pl` (see Appendix A.3) on the fifth field of raw base calls to mask out adjacent indels with an `X` character and remove the references to mapping quality (`^.`) that follow base calls at within-read position one. We retained the `$` characters that follow base calls at within-read position 100 (end of read), and later had to work around these in `pileupsummary.R`, a script for summarising the information contained in each pileup file (Appendix A.4).

The script `buildsummarydataframe.R` for extracting and reformatting information from the four algorithms' native output formats and combining this with query results from dbSNP build 137 (using BioQ, Saccone et al. (2012)) and the pileup summaries to generate one consistent and informative data frame for the candidate sites is provided in Appendix A.5. Table 2 describes the variables defined by `pileupsummary.R` and `buildsummarydataframe.R`.

2 Filtering results

First, we defined a filter to remove sites with 100% strand bias, essentially requiring that each candidate site have at least one variant base from each strand, as summarised in Table 3.

After applying the strand bias filter, we defined five additional filters at conservative thresholds designed to remove only the most unconvincing candidates. We applied each filter separately to the output, with strand bias already removed, to analyse their individual effects.

Table 4 summarises the removal of sites with more than two candidate SNVs (identified by any algorithm) within 50 bp either side. This filter is designed to remove phasing sequencing errors and poorly mapped reads with multiple mismatches. Overall, 92.4% of sites passed, with JSM2 having the lowest proportion pass and Strelka the highest.

Table 5 summarises the removal of sites with spanning deletions contributing more than 20% to the overall depth in either the cancer or the normal sample. This filter is designed to remove sites in dubiously mapped regions, such as those existing at repetitive or common sequences. Overall, 97.4% of sites passed, with SomaticSniper and JSM2 having the lowest proportion pass and Strelka and VarScan having the highest.

Table 6 summarises the removal of sites immediately adjacent to indels in more than 20% of reads in either cancer or normal. This filter is designed to remove sites that differ from reference by virtue of the mapping algorithm for indel placement, though this comes at the cost of missing any real somatic sites genuinely adjacent to an indel event. Overall, 97.3% of sites passed, with JSM2 having the lowest proportion pass and VarScan the highest.

Table 7 summarises the removal of sites with poor variant base quality, considering variants of the cancer sample for somatic candidates and normal sample for LOH candidates. Sites are retained if the mean variant base quality is at least 15 and at least one variant has base quality 30 or over. Overall, 98.8% of sites passed, with JSM2 having the lowest proportion pass and SomaticSniper the highest.

[2] `samtools mpileup -B -O -s -l sites.txt -f ref.fasta input.bam > out.pileup`

Table 2: Variable names and definitions, in the files some variable names have further extensions to identify the sample to which they refer

chr	chromosome
pos	base position
ref	reference base
titv	transition (ti) or transversion (tv)
state	gain of variation in the cancer (S) or loss of heterozygosity (L)
vs	probability of somatic mutation output by VarScan, NA if not called
ss	probability of somatic mutation output by SomaticSniper, NA if not called
js	probability of somatic mutation output by JointSNVMix2, NA if not called
st	probability of somatic mutation output by Strelka, NA is not called
stfilt	result of Strelka's post-calling filtration
dbSNPrs	rs number of the first entry in dbSNP for this site, NA if not present
nearsnps	number of SNV calls within ± 50 bases, with a limit of 20
hits	number of algorithms returning the site
consensus	the average somatic probability of the algorithms returning the site
depth	total number of bases
refcount	number of reference bases
refprop	proportion of reference bases
acount	number of variant 'A' bases
ccount	number of variant 'C' bases
gcount	number of variant 'G' bases
tcount	number of variant 'T' bases
varcount	total number of variant bases
varprop	proportion of variant bases
ncount	number of 'N' base calls
adjindel	number of adjacent indels
readdel	number of reads spanning deletions
varforstr	number of variants on the forward strand
varrevstr	number of variants on the reverse strand
varstbias	percentage of variant reads on the preferred strand
rbqmean	mean base quality of reference bases
rmqmean	mean mapping quality of reference bases
vbqmean	mean base quality of variant bases
vbqmax	maximum base quality of variant bases
vmqmean	mean mapping quality of variant bases
vmqmax	maximum mapping quality of variant bases
reprop	proportion of reference bases within first 5 positions
rlprop	proportion of reference bases within last 10 positions
veprop	proportion of variant bases within first 5 positions
vlprop	proportion of variant bases within last 10 positions
varsamepos	highest number of variant bases in the same within-read position

Table 3: Strand bias filter on raw CML output

		Pass	Fail	%Pass	Significance
Multiple callers?	No	4307	4933	46.61	2.0×10^{-13}
	Yes	694	504	57.93	***
In dbSNP?	No	1949	2491	43.90	1.9×10^{-12}
	Yes	3052	2946	50.88	***
Algorithm	VS	2207	2180	50.31	overall
	SS	1430	1408	50.39	$< 2.2 \times 10^{-16}$
	JSM	1930	2288	45.76	pairwise: all except
	ST	326	134	70.87	VS with SS

Table 4: Nearby SNV filter on CML exome at diagnosis

		Pass	Fail	%Pass	Significance
Multiple callers?	No	4006	301	93.01	2.0×10^{-4}
	Yes	617	77	88.90	***
In dbSNP?	No	1838	11	94.30	8.5×10^{-5}
	Yes	2785	267	91.25	***
Algorithm	VS	2063	144	93.48	overall
	SS	1316	114	92.03	3.9×10^{-12}
	JSM	1715	215	88.86	pairwise: all except
	ST	324	2	99.39	VS with SS

Table 5: Spanning deletions filter on CML at exome at diagnosis

		Pass	Fail	%Pass	Significance
Multiple callers?	No	4224	83	98.07	5.9×10^{-14}
	Yes	646	48	93.08	***
In dbSNP?	No	1918	31	98.41	3.9×10^{-4}
	Yes	2952	100	96.72	***
Algorithm	VS	2187	20	99.09	overall
	SS	1365	65	95.45	$< 2.2 \times 10^{-16}$
	JSM	1831	99	94.87	pairwise: all except
	ST	324	2	99.39	SS with JS; VS with ST

Table 6: Adjacent indel filter on CML exome at diagnosis

		Pass	Fail	%Pass	Significance
Multiple callers?	No	4211	96	97.77	1.7×10^{-6}
	Yes	656	38	94.52	***
In dbSNP?	No	1901	48	97.54	0.50
	Yes	2966	86	97.18	
Algorithm	VS	2206	1	99.95	overall
	SS	1374	56	96.08	$< 2.2 \times 10^{-16}$
	JSM	1820	110	94.30	pairwise: VS
	ST	319	7	97.85	with all others

Table 7: Variant base quality filter on CML exome at diagnosis

		Pass	Fail	%Pass	Significance
Multiple callers?	No	4249	58	98.65	0.064
	Yes	691	3	99.57	
In dbSNP?	No	1903	46	97.64	9.5×10^{-9}
	Yes	3037	15	99.51	***
Algorithm	VS	2184	23	98.96	overall (simulated)
	SS	1424	6	99.58	0.004
	JSM	1897	33	98.29	pairwise: only
	ST	324	2	99.39	SS with JS

Table 8: Variant mapping quality filter on CML exome at diagnosis

		Pass	Fail	%Pass	Significance
Multiple callers?	No	2857	1450	66.33	0.009
	Yes	496	198	71.47	*
In dbSNP?	No	1377	572	70.65	1.7×10^{-5}
	Yes	1976	1076	64.74	***
Algorithm	VS	1373	834	62.21	overall
	SS	1107	323	77.41	$< 2.2 \times 10^{-16}$
	JSM	1227	703	63.58	pairwise: all except
	ST	305	21	93.56	VS with JS

Table 8 summarises the removal of sites with poor variant mapping quality, considering variants of the cancer sample for somatic candidates and normal sample for LOH candidates. Sites are retained if the mean variant mapping quality is at least 15 and at least one variant has mapping quality 40 or over. Overall, 67.0% of sites passed this filter, with VarScan and JSM2 having the lowest proportions of 62.21% and 63.58% pass respectively, compared to SomaticSniper with 77.41% and Strelka with 93.56% pass rate.

Finally, these five additional filters were applied together to the output, with strand bias already removed, as summarised in Table 9. There were significant differences between each algorithm, with 47.93% of JSM2 sites passing all filters, compared with 58.31% of VarScan sites, 66.22% of SomaticSniper sites and 89.88% of Strelka sites.

Table 9: Filters for nearby SNVs, spanning deletions, adjacent indels, and variant base and mapping qualities applied together to CML exome at diagnosis, with strand bias already removed

		Pass	Fail	%Pass	Significance
Multiple callers?	No	2535	1772	58.86	0.102
	Yes	385	309	55.48	
In dbSNP?	No	1217	732	62.44	3.9×10^{-6}
	Yes	1703	1349	55.80	***
Algorithm	VS	1287	920	58.31	overall
	SS	947	483	66.22	$< 2.2 \times 10^{-16}$
	JSM	925	1005	47.93	pairwise: all
	ST	293	33	89.88	

References

Koboldt, D. C., Chen, K., Wylie, T., Larson, D. E., McLellan, M. D., Mardis, E. R., Weinstock, G. M., Wilson, R. K. & Ding, L. (2009), 'VarScan: variant detection in massively parallel sequencing of individual and pooled samples', *Bioinformatics* **25**(17), 2283–2285. `http://bioinformatics.oxfordjournals.org/content/25/17/2283.abstract`

Koboldt, D., Zhang, Q., Larson, D., Shen, D., McLellan, M., Lin, L., Miller, C., Mardis, E., Ding, L. & Wilson, R. (2012), 'VarScan 2: Somatic mutation and copy number alteration discovery in cancer by exome sequencing.', *Genome Res* **22**, 568–576.

Larson, D. E., Harris, C. C., Chen, K., Koboldt, D. C., Abbott, T. E., Dooling, D. J., Ley, T. J., Mardis, E. R., Wilson, R. K. & Ding, L. (2012), 'SomaticSniper: identification of somatic point mutations in whole genome sequencing data', *Bioinformatics* **28**(3), 311–317. `http://bioinformatics.oxfordjournals.org/content/28/3/311.abstract`

Li, H., Handsaker, B., Wysoker, A., Fennell, T., Ruan, J., Homer, N., Marth, G., Abecasis, G. & Durbin, R. (2009), 'The Sequence Alignment/Map format and SAMtools.', *Bioinformatics* **25**(16), 2078–2079.

Roth, A., Ding, J., Morin, R., Crisan, A., Ha, G., Giuliany, R., Bashashati, A., Hirst, M., Turashvili, G., Oloumi, A., Marra, M. A., Aparicio, S. & Shah, S. P. (2012), 'JointSNVMix: a probabilistic model for accurate detection of somatic mutations in normal/tumour paired next-generation sequencing data', *Bioinformatics* **28**(7), 907–913. `http://bioinformatics.oxfordjournals.org/content/28/7/907.abstract`

Saccone, S. F., Quan, J. & Jones, P. L. (2012), 'BioQ: tracing experimental origins in public genomic databases using a novel data provenance model', *Bioinformatics* **28**(8), 1189–1191. `http://bioinformatics.oxfordjournals.org/content/28/8/1189.abstract`

Saunders, C. T., Wong, W. S. W., Swamy, S., Becq, J., Murray, L. J. & Cheetham, R. K. (2012), 'Strelka: accurate somatic small-variant calling from sequenced tumor–normal sample pairs', *Bioinformatics* **28**(14), 1811–1817. `http://bioinformatics.oxfordjournals.org/content/28/14/1811.abstract`

A Scripts

This code is also available at `http://code.google.com/p/snv-caller-review/`

A.1 halvebam.go

```
package main

import (
	"code.google.com/p/biogo.boom"

	"flag"
	"fmt"
	"math/rand"
	"os"
	"time"
)

var (
	in, out string
	seed    int64
	paired  bool
)

func init() {
	flag.StringVar(&in, "in", "", "Infile name - must be name sorted.")
	flag.StringVar(&out, "out", "", "Outfile name.")
	flag.Int64Var(&seed, "seed", -1, "Random seed (<0 for time seeded).")
```

```go
	flag.BoolVar(&paired, "paired", true, "Reads are paired.")

	flag.Parse()
	if in == "" || out == "" {
		flag.Usage()
		panic("Bad")
	}

	if seed < 0 {
		seed = time.Now().Unix()
	}
	fmt.Printf("Using %d as seed\n.", seed)
	rand.Seed(seed)
}

func main() {
	boom.Verbosity(0)

	// Read in BAM file to bf.
	bf, err := boom.OpenBAM(in)
	if err != nil {
		panic(err)
	}

	var (
		setName = []string{"tum-", "norm-"}
		bo      [2]*boom.BAMFile
	)

	// Open two new empty BAM files to be "tumour" and "normal".
	for i := 0; i < 2; i++ {
		bo[i], err = boom.CreateBAM(setName[i]+out, bf.Header(), true)
		if err != nil {
			panic(err)
		}
		defer bo[i].Close()
	}

	for {
		// Set is randomly allocated 0 or 1.
		set := rand.Int31n(2)
		var r2 *boom.Record
		// r1 is the next read of the input BAM.
		r1, _, err := bf.Read()
		if err != nil {
			break
		}
		if paired {
			// r2 is the paired read of r1.
			r2, _, err = bf.Read()
			if err != nil {
				break
			}
			if r1.Name() != r2.Name() {
				fmt.Fprintln(os.Stderr, r1.Name(), r2.Name())
				panic("name mismatch")
			}
		}
```

```

        _, err = bo[set].Write(r1)
        if err != nil {
            fmt.Fprintln(os.Stderr, err)
        }

        if paired {
            // Allocate r1 (and r2 if paired) to the tumour
            // or normal output BAM files as indicated by
            // the value of set.
            _, err = bo[set].Write(r2)
            if err != nil {
                fmt.Fprintln(os.Stderr, err)
            }
        }
    }
}
```

A.2 snvcall.sh

```
#!/bin/bash
set -v
nice samtools index HSS41_GATK.bam &
nice samtools index HSS43_GATK.bam &
wait
nice bam-somaticsniper -q 1 -Q 3 -J -s 0.0001 -f hs37d5.fa HSS41_GATK.bam HSS43_GATK.bam ssp1diagraw &
nice samtools mpileup -q 1 -f hs37d5.fa HSS41_GATK.bam > HSS41_GATK.pileup &
nice samtools mpileup -q 1 -f hs37d5.fa HSS43_GATK.bam > HSS43_GATK.pileup &
nice jsm.py train joint_snv_mix_two hs37d5.fa HSS43_GATK.bam HSS41_GATK.bam /media/Data/JointSNVMix-0.7.5/config/joint_priors.cfg /media/Data/JointSNVMix-0.7.5/config/joint_params.cfg jsm2p1diagparams &
wait
nice jsm.py classify joint_snv_mix_two hs37d5.fa HSS43_GATK.bam HSS41_GATK.bam jsm2p1diagparams jsm2p1rawcalls &
nice java -jar /media/Data/VarScan.v2.2.11.jar somatic HSS43_GATK.pileup HSS41_GATK.pileup vsp1diag --somatic-p-value 0.5 --strand-filter 1 &
wait
nice java -jar /media/Data/VarScan.v2.2.11.jar processSomatic vsp1diag.snp &
wait
nice awk '$9<=0.5' jsm2p1rawcalls > jsm2p1soi.txt &
nice /media/Data/strelka_workflow/configureStrelkaWorkflow.pl --normal=HSS43_GATK.bam --tumor=HSS41_GATK.bam --ref=hs37d5.fa --config=config.ini --output-dir=./StrelkaP1 &
wait
```

A.3 cleanpileup.pl

```
#!/usr/bin/perl
my $file = $ARGV[0];
open MYFILE, "<", $file;
```

```
while (<MYFILE>){
  s/\^.//g; #this removes references to read start qualities
  while (m/[+-]([0-9]+)/){
    s/[+-]$1[ACGTNacgtn]{$1}/X/g
    }; #this masks adjacent indels with a X
  print "$_";
};
```

A.4 pileupsummary.R

```
pilesummary <- function(x){
    chr <- x[,1]
    pos <- x[,2]
    ref <- x[,3]
    depth <- x[,4]

    bcstr <- sapply(x[,5], toString)
    bc <- sapply(bcstr, strsplit, split=NULL, USE.NAMES=FALSE)  #vector of basecalls
    refcount <- sapply(sapply(bc, grep, pattern="[\\.|\\,]"), length) #number matching reference
    refprop <- refcount/depth #proportion matching reference
    acount <- sapply(sapply(bc, grep, pattern="[a|A]"), length) #variant A bases
    ccount <- sapply(sapply(bc, grep, pattern="[c|C]"), length) #variant C bases
    gcount <- sapply(sapply(bc, grep, pattern="[g|G]"), length) #variant G bases
    tcount <- sapply(sapply(bc, grep, pattern="[t|T]"), length) #variant T bases
    varcount <- sapply(sapply(bc, grep, pattern="[a|A|c|C|g|G|t|T]"), length) #number of variant bases
    varprop <- varcount/depth #proportion of variant bases
    ncount <- sapply(sapply(bc, grep, pattern="[n|N]"), length) #number of unknown bases
    adjindel <- sapply(sapply(bc, grep, pattern="X"), length) #number of adjacent indels inf the alignment
    readdel <- sapply(sapply(bc, grep, pattern="\\*"), length) #number of reads with deletions spanning the site

    varforstr <- sapply(sapply(bc, grep, pattern="[A|C|G|T]"), length) #number of variants from forward strand
    varrevstr <- sapply(sapply(bc, grep, pattern="[a|c|g|t]"), length) #number of variants from reverse strand
     stbias <- function(ford, revs){
            b <- max(ford, revs)/sum(ford, revs)
            return(b)}
    varstbias <- mapply(stbias, varforstr, varrevstr) #percentage of variant reads coming from the preferred strand

    f0 <- function(y){out <- y-33;return(out)} #function to adjust ASCII numerical code by -33

    bqstr <- sapply(x[,6], toString) #base qualities as ASCII string
    bqoff <- sapply(sapply(bqstr, charToRaw), as.numeric) #numeric base qualities, offset by 33
```

```
bq <- sapply(bqoff, f0) #correct base qualities

mqstr <- sapply(x[,7], toString) #mapping qualities as ASCII string
mqoff <- sapply(sapply(mqstr, charToRaw), as.numeric) #numeric mapping qualities, offset by 33
mq <- sapply(mqoff, f0) #correct mapping qualities

rpstr <- sapply(x[,8], toString)
rpspl <- sapply(rpstr, strsplit, split=",", USE.NAMES=FALSE)
rp <- sapply(rpspl, as.numeric) #vector of within read positions.

    rbqmean <- rep(NA, nrow(x))
    rmqmean <- rep(NA, nrow(x))
    rearly <- rep(NA, nrow(x))
    rlate <- rep(NA, nrow(x))
    vbqmean <- rep(NA, nrow(x))
    vbqmax <- rep(NA, nrow(x))
    vmqmean <- rep(NA, nrow(x))
    vmqmax <- rep(NA, nrow(x))
    vearly <- rep(NA, nrow(x))
    vlate <- rep(NA, nrow(x))
    varsamepos <- rep(NA, nrow(x))

    for (i in 1:nrow(x)) {
        calls <- bc[[i]][which(bc[[i]]!="$" & bc[[i]]!="X")] #extract actual base calls from bc, bc includes adjacent indel maskings (X) and indicators of read ends ($). Leave in *'s for spanning deletions because read positions, mapping qualities and base qualities are also assigned to *'s.
        if (refcount[i]==0) {refpos <- NaN} else{refpos <- which(calls %in% c(".", ","))}
        if (varcount[i]==0) {varpos <- NaN} else{varpos <- which(calls %in% c("a","A","c","C","g","G","t","T"))}
        #which positions along 'calls' contain the reference and variant bases.

        refbq <- bq[[i]][refpos] #base qualities of reference bases
        refmq <- mq[[i]][refpos] #mapping qualities of reference bases
        refrp <- rp[[i]][refpos] #within read positions of reference bases
        varbq <- bq[[i]][varpos] #base qualities of variant bases
        varmq <- mq[[i]][varpos] #mapping qualities of variant bases
        varrp <- rp[[i]][varpos] #within read positions of variant bases

        rbqmean[i] <- mean(refbq) #mean base quality of reference bases
        rmqmean[i] <- mean(refmq) #mean mapping qualitiy of reference bases
        rearly[i] <- length(which(refrp <= 5)) # number of reference bases within first 5 positions
        rlate[i] <- length(which(refrp > 90)) #number of reference bases within last 10 positions
        vbqmean[i] <- mean(varbq) #mean base quality of variant bases
        vbqmax[i] <- max(varbq) #maximum base quality of variant bases
        vmqmean[i] <- mean(varmq) #mean mapping quality of variant bases
        vmqmax[i] <- max(varmq) #maximum mapping quality of variant bases
```

```
        vearly[i] <- length(which(varrp <= 5)) #number of variant bases within first 5 positions
        vlate[i] <-  length(which(varrp > 90)) #number of variant bases within last 10 positions.
        if (is.na(varpos)[1]) {varsamepos[i]=0} else {varsamepos[i] <- max(table(varrp)) } #maximum number of variant bases with a shared position. Can indicate variants from PCR duplicates.
    }

  reprop <- rearly/refcount #proportion of reference bases within first 5 positions
  rlprop <- rlate/refcount #proportion of reference bases within last 10 positions
  veprop <- vearly/varcount #proportion of variant bases within first 5 positions
  vlprop <- vlate/varcount #proportion of variant bases within last 10 positions

  return(data.frame(chr, pos, ref, depth, refcount, refprop, acount, ccount, gcount, tcount, varcount, varprop, ncount, adjindel, readdel, varforstr, varrevstr, varstbias, rbqmean, rmqmean, vbqmean, vbqmax, vmqmean, vmqmax, reprop, rlprop, veprop, vlprop, varsamepos, row.names=NULL))
}

pile41 <- read.delim("HSS41_cleansites.pileup", header=FALSE, sep="\t", quote="", dec=NULL)
pile43 <- read.delim("HSS43_cleansites.pileup", header=FALSE, sep="\t", quote="", dec=NULL)

summary41 <- pilesummary(pile41)
write.table(summary41, file="pilesummary41.txt", quote=FALSE, sep="\t", row.names=FALSE)
summary43 <- pilesummary(pile43)
write.table(summary43, file="pilesummary43.txt", quote=FALSE, sep="\t", row.names=FALSE)
```

A.5 buildsummarydataframe.R

```
library(stringr)

#read in output from somatic SNV callers

vsdiagsom <- read.table("vsp1diag.snp.Somatic", header=TRUE)
vsdiagloh <- read.table("vsp1diag.snp.LOH", header=TRUE)

vsdiagmutn <- rbind(vsdiagsom, vsdiagloh)
vsdiagmut <- vsdiagmutn[order(vsdiagmutn$chrom, vsdiagmutn$pos),]

sscols <- c("chr", "pos", "ref", "IUBt", "IUBn", "somscore", "tconqual", "tvarqual", "tmapqual", "nconqual", "nvarqual", "nmapqual", "tdep", "ndep", "trefbq", "trefmq", "trefdep", "tvarbq", "tvarmq", "tvardep", "nrefbq", "nrefmq", "nrefdep", "nvarbq", "nvarmq", "nvardep")
ssdiag <- read.table("ss1diag", header=FALSE)
names(ssdiag)<- sscols
```

```
jsm2diagsom50 <- read.table("jsm2p1diagsom50", header=TRUE)
jsm2diagloh50 <- read.table("jsm2p1diagloh50", header=TRUE)

jsm2diagmut50 <- rbind(jsm2diagsom50, jsm2diagloh50)

stdiagall <- read.table("stp1diag.all.som.snvs", header=FALSE)


#function to convert phred scale quality score to probability
dephred <- function(q){
    p <- 1-10^(-q/10)
    return(p)
}

#function to extract the somatic mutation probability from raw Strelka
    output
extractqual <- function(field){
    a <- sapply(field, toString)
    b <- sapply(a, str_extract, pattern="QSS_NT=[0-9]+", USE.NAMES=FALSE)
    c <- sapply(b, str_extract, pattern="[0-9]+", USE.NAMES=FALSE)
    d <- as.numeric(c)
    return(d)
}

#function to identify transitions and transversions from simple reference
    and variant base info
titv <- function(ref, var){
    titv <- c()
    for (i in 1:length(ref)){
    if (ref[i]=="A" & var[i]=="G" | ref[i]=="G" & var[i]=="A" | ref[i]=="C"
        & var[i]=="T" |ref[i]=="T" & var[i]=="C") {t="ti"}
    else {t="tv"}
    titv = c(titv, t)
    }
    return(titv)
}

#function to identify transitions and transversions from IUB genotype codes
     (for SomaticSniper output)
titvIUB <- function(IUBn, IUBt){
    titv <- c()
    for (i in 1:length(IUBn)){
    if (IUBn[i]=="A" & IUBt[i] %in% c("G", "R") | IUBn[i]=="G" & IUBt[i] %
       in% c("A", "R") | IUBn[i]=="C" & IUBt[i] %in% c("T", "Y") | IUBn[i
       ]=="T" & IUBt[i] %in% c("C", "Y") | IUBn[i]=="R" & IUBt[i] %in% c("A
       ", "G") | IUBn[i]=="Y" & IUBt[i] %in% c("C", "T") | IUBn[i]=="K" &
       IUBt[i] %in% c("W", "S") | IUBn[i]=="M" & IUBt[i] %in% c("S", "W") |
        IUBn[i]=="S" & IUBt[i] %in% c("K", "M") | IUBn[i]=="W" & IUBt[i] %
       in% c("K", "M")) {t="ti"}
    else {t="tv"}
    titv = c(titv, t)
    }
    return(titv)
}

#function to identify somatic and LOH states from VarScan output
statusVS <- function(sl){
    status <- c()
```

```r
    for (i in 1:length(sl)) {
        if (sl[i]=="Somatic") {s="S"}
        else {s="L"}
        status <- c(status, s)
    }
    return(status)
}

#function to identify somatic and LOH states from SomaticSniper output
statusSS <- function(IUBn, IUBt){
    status <- c()
    for (i in 1:length(IUBn)) {
        if (IUBn[i] %in% c("R", "Y", "K", "M", "S", "W") & IUBt[i] %in% c("A", "C", "G", "T")) {s="L"}
        else {s="S"}
        status <- c(status, s)
    }
    return(status)
}

#function to identify somatic and LOH states from JSM output
statusJSM <- function(somprob, lohprob){
        status <- c()
        for (i in 1:length(somprob)) {
        if (somprob[i] > lohprob[i]) {s="S"}
        else {s="L"}
        status <- c(status, s)
    }
    return(status)
}

#function to identify somatic and LOH states from Strelka output
statusST <- function(field) {
    a <- sapply(field, toString)
    b <- sapply(a, str_extract, pattern="(ref)|(het)|(hom)", USE.NAMES=FALSE)
    status <- c()
    for (i in 1:length(field)) {
        if (b[i] %in% c("ref", "hom")) {s="S"}
        else {s="L"}
        status <- c(status, s)
        }
    return(status)
}

#function to calcualte the number of SNV calls within 50bp either side of
    each site, the number of algorithms returning the same site (hits), and
    the average (consensus) somatic probability of the site
nearhitscon <- function(x){
    calls <- x[,5:8]
    ns <- rep(-1, nrow(x))
    h <-rep(1, nrow(x))
    con <- rep(0, nrow(x))
    for (i in 1:nrow(x)){
        #how many SNPs within 50bp
        chr = x[i,1]
        pos = x[i,2]
        range = c(pos-50, pos+50)
```

```
        for (j in max(1,(i-10)):min((i+10),nrow(x))) {
            if (x[j,1]==chr & range[1] <= x[j,2] & x[j,2] <= range[2]) {ns[i] <- ns[i]+1}
        }
        poscols <- which(calls[i,]!="NA")
        con[i] <- sum(calls[i,poscols])/length(poscols)
        h[i] <- length(poscols)
    }
    return(data.frame(nearsnps=ns, hits=h, consensus=con))
}

#extract the position, ti/tv state, somatic probability, and somatic/LOH state for sites from all four callers, and the filter field for Strelka's output as well

vsdsites <- data.frame(chr=vsdiagmut[,1], pos=vsdiagmut[,2], titvd=titv(vsdiagmut[,3], vsdiagmut[,4]), vsdiag=round((1-vsdiagmut$somatic_p_value), digits=5), stated=statusVS(vsdiagmut$somatic_status))

ssdsites <- data.frame(chr=ssdiag[,1], pos=ssdiag[,2], titvd=titvIUB(ssdiag[,5], ssdiag[,4]), ssdiag=round(dephred(ssdiag$somscore), digits=3), stated=statusSS(ssdiag$IUBn, ssdiag$IUBt))

jsdsites <- data.frame(chr=jsm2diagmut50[,1], pos=jsm2diagmut50[,2], titvd=titv(jsm2diagmut50[,3], jsm2diagmut50[,4]), jsdiag=round(mapply(max, jsm2diagmut50$somprob, jsm2diagmut50$lohprob),digits=5), stated=statusJSM(jsm2diagmut50$somprob, jsm2diagmut50$lohprob))

stdsites <- data.frame(chr=stdiagall[,1], pos=stdiagall[,2], titvd=titv(stdiagall[,4], stdiagall[,5]), stdiag=round(dephred(extractqual(stdiagall[,8])), digits=3), stated=statusST(stdiagall$V8), stdfilt=stdiagall[,7])

#merge site results for VarScan and SomaticSniper output and resolve differences in stated and titvd field so that each site has one row

da <- merge(vsdsites, ssdsites, all=TRUE)

da[c(rbind(which(duplicated(da[,1:2])==TRUE)-1, which(duplicated(da[,1:2])==TRUE))),]

chuckda <- c()
for(i in 1:length(which(duplicated(da[,1:2])==TRUE))){
    row2 <- which(duplicated(da[,1:2])==TRUE)[i]
    row1 <- row2-1
    #if the S/L state disagrees, remove the line with lowest prob.
    if (da[row1,4]!=da[row2,4]) {
        m <- which.max(c(da[row1,5], da[row1,6], da[row2,5], da[row2,6]))
        if (m %in% c(1,2)) {
            chuckda <- c(chuckda, row2)
            } else {
                chuckda <- c(chuckda, row1)
            }
    }
    #if the ti/tv disagrees, go with SS call, put both probs in that row, and remove the other row.
    else {
        ssrow <- which(c(da[row1,6], da[row2,6])>0)
```

```
        if (ssrow==1) {
            da[row1,5]=da[row2,5]
            chuckda <- c(chuckda, row2)
            } else {
                da[row2,5]=da[row1,5]
                chuckda <- c(chuckda, row1)
                }
            }
}

da2 <- da[which(!c(1:nrow(da)) %in% chuckda),]

#merge with JSM2 output and resolve differences in stated and titvd field so that each site has one row

db <- merge(da2, jsdsites, all=TRUE)

db[c(rbind(which(duplicated(db[,1:2])==TRUE)-1, which(duplicated(db[,1:2])==TRUE))),]

chuckdb <- c()
for(i in 1:length(which(duplicated(db[,1:2])==TRUE))){
    row2 <- which(duplicated(db[,1:2])==TRUE)[i]
    row1 <- row2-1
    #if the S/L state disagrees, remove the line with lowest prob.
    if (db[row1,4]!=db[row2,4]) {
        m <- which.max(c(db[row1,5], db[row1,6], db[row1,7], db[row2,5], db[row2,6], db[row2,7]))
        if (m %in% c(1,2,3)) {
            chuckdb <- c(chuckdb, row2)
            } else {
                chuckdb <- c(chuckdb, row1)
            }
    }
    #if the ti/tv disagrees, go with SS call, put both probs in that row, and remove the other row.
    else {
        ssrow <- which(c(db[row1,6], db[row2,6])>0)
        if (ssrow==1) {
            db[row1,7]=db[row2,7]
            chuckdb <- c(chuckdb, row2)
            } else {
                db[row2,7]=db[row1,7]
                chuckdb <- c(chuckdb, row1)
                }
            }
}


db2 <- db[which(!c(1:nrow(db)) %in% chuckdb),]

#merge with Strelka output and resolve differences in stated and titvd field so that each site has one row

dc <- merge(db2, stdsites, all=TRUE)

chuckdc <- c()
for(i in 1:length(which(duplicated(dc[,1:2])==TRUE))){
```

```
    row2 <- which(duplicated(dc[,1:2])==TRUE)[i]
    row1 <- row2-1
    #if the S/L state disagrees, remove the line with lowest prob.
    if (dc[row1,4]!=dc[row2,4]) {
        m <- which.max(c(dc[row1,5], dc[row1,6], dc[row1,7], dc[row1,8], dc
            [row2,5], dc[row2,6], dc[row2,7], dc[row2,8]))
        if (m %in% c(1,2,3,4)) {
            chuckdc <- c(chuckdc, row2)
            } else {
                chuckdc <- c(chuckdc, row1)
            }
    }
    #if the ti/tv disagrees, go with SS call, put both probs in that row,
        and remove the other row.
    else {
        ssrow <- which(c(dc[row1,6], dc[row2,6])>0)
        if (ssrow==1) {
            dc[row1,8]=dc[row2,8]
            chuckdc <- c(chuckdc, row2)
            } else {
                dc[row2,8]=dc[row1,8]
                chuckdc <- c(chuckdc, row1)
                }
            }
}


dc2 <- dc[which(!c(1:nrow(dc)) %in% chuckdc),]

#write out a query for BioQ::Query dbSNP.137, read in the results, for
    sites with more than one dbSNP rs number, keep only the first one, and
    merge dbSNP results with existing dataframe

dbSNPQ <- function(sites){
    out <- c()
    chrom <- sites[,1]
    pos <- sites[,2]
    for (i in 1:nrow(sites)){
        string <- paste("REGION=Chr", chrom[i], ":", pos[i], "..", pos[i],
            sep="")
    out <- c(out, string)
    }
    matrix <- matrix(out, ncol=1)
    return(matrix)
}

dbSNPQueryD <- dbSNPQ(dc2)
write.table(dbSNPQueryD, file="dbSNPQueryD.txt", quote=FALSE, row.names=
    FALSE, col.names=FALSE)
#==go to http://bioq.saclab.net/query/submit.php?db=bioq_dbsnp_human_137
#ran SNP summary query with max rows=50000 and saved raw result as
    dbSNPquery_resultD.txt
#cut -f 2,5,6 dbSNPquery_resultsD.txt > dbSNPquerysummaryD.txt

dbsnpqD <- read.table("dbSNPquerysummaryD.txt", header=TRUE)
names(dbsnpqD) <- c("dbSNPrs", "chr", "pos")
dbsnpfirstD <- row.names(unique(dbsnpqD[,2:3]))
dbsnpD <- dbsnpqD[dbsnpfirstD,]
```

```r
dd <- merge(dc2, dbsnpD all.x=TRUE)


#calculate number of nearby SNPS, number of 'hits' and the consensus somatic probability and add to dataframe

dnearhitscon <- nearhitscon(dd)

de <- data.frame(dd, dnearhitscon)
names(de) <- c(names(de)[1:10], "dnearsnps", "dhits", "dconsensus")

#read in pileup summary files (see pileupsummary.R) and add to dataframe

pile41 <- read.table("pilesummary41.txt", header=TRUE)
oldnames <- names(pile41)
newnames41 <- c(oldnames[1:3], paste(oldnames[4:29], 41, sep=""))
names(pile41) <- newnames41

pile43 <- read.table("pilesummary43.txt", header=TRUE)
newnames43 <- c(oldnames[1:3], paste(oldnames[4:29], 43, sep=""))
names(pile43) <- newnames43

df <- merge(de, pile43)
dg <- merge(df, pile41)

#write out final dataset for the CML exome at diagnosis

write.table(dg, file="P1DIAGcandidatesites.txt", quote=FALSE, row.names=FALSE, col.names=TRUE)
```
